# Broad Serotonergic Actions of Vortioxetine as a Promising Avenue for the Treatment of L-DOPA-Induced Dyskinesia

**DOI:** 10.3390/cells12060837

**Published:** 2023-03-08

**Authors:** Carla Budrow, Kayla Elder, Michael Coyle, Ashley Centner, Natalie Lipari, Sophie Cohen, John Glinski, N’Senga Kinzonzi, Emily Wheelis, Grace McManus, Fredric Manfredsson, Christopher Bishop

**Affiliations:** 1 Behavioral Neuroscience Program, Department of Psychology, Binghamton University, Binghamton, NY 13902, USA; cbudrow1@binghamton.edu (C.B.);; 2 Department of Neurobiology, Barrow Neurological Institute, Phoenix, AZ 85013, USA

**Keywords:** levodopa-induced dyskinesia, Parkinson’s disease, serotonin, dopamine, serotonin transporter, 5-HT_1A_ receptor, 5-HT_1B_ receptor, vortioxetine, 6-hydroxydopamine

## Abstract

Parkinson’s Disease (PD) is a neurodegenerative disorder characterized by motor symptoms that result from loss of nigrostriatal dopamine (DA) cells. While L-DOPA provides symptom alleviation, its chronic use often results in the development of L-DOPA-induced dyskinesia (LID). Evidence suggests that neuroplasticity within the serotonin (5-HT) system contributes to LID onset, persistence, and severity. This has been supported by research showing 5-HT compounds targeting 5-HT_1A/1B_ receptors and/or the 5-HT transporter (SERT) can reduce LID. Recently, vortioxetine, a multimodal 5-HT compound developed for depression, demonstrated acute anti-dyskinetic effects. However, the durability and underlying pharmacology of vortioxetine’s anti-dyskinetic actions have yet to be delineated. To address these gaps, we used hemiparkinsonian rats in Experiment 1, examining the effects of sub-chronic vortioxetine on established LID and motor performance. In Experiment 2, we applied the 5-HT_1A_ antagonist WAY-100635 or 5-HT_1B_ antagonist SB-224289 in conjunction with L-DOPA and vortioxetine to determine the contributions of each receptor to vortioxetine’s effects. The results revealed that vortioxetine consistently and dose-dependently attenuated LID while independently, 5-HT_1A_ and 5-HT_1B_ receptors each partially reversed vortioxetine’s effects. Such findings further support the promise of pharmacological strategies, such as vortioxetine, and indicate that broad 5-HT actions may provide durable responses without significant side effects.

## 1. Introduction

Parkinson’s Disease (PD) is a debilitating neurodegenerative disease characterized by bradykinesia, tremor, rigidity, and postural instability [1] resulting from progressive dopamine (DA) cell loss in the substantia nigra [2,3]. As the second most prevalent neurodegenerative disorder, PD affects as many as 6 million individuals worldwide, an increase that has doubled over the last 25 years [4]. Fortunately, DA replacement therapy with DA precursor L-3,4-dihydroxyphenylalanine or levodopa (L-DOPA) effectively attenuates motor symptoms in early-stage PD [5]. Regrettably, within 10 years of chronic treatment, upwards of 90% of patients experience drug-related choreic and dystonic movements termed L-DOPA-induced dyskinesia (LID; [6,7]). LID can have detrimental effects on patient and caregiver quality of life while introducing additional economic stressors [8,9,10]. Given the dearth of effective treatments, unraveling the neurobiological basis of LID and investigating novel approaches that reduce LID remain paramount.

Recent studies point to a compensatory role for the serotonin (5-HT) system in mediating L-DOPA-driven DA release in later stages of PD in states of severe nigrostriatal depletion. Striatal 5-HT neuronal hyperinnervation has been demonstrated in L-DOPA-treated PD patients [11,12] and has been replicated in DA-denervated non-human primates and rodents [13,14]. Clinical investigations have positively correlated SERT:DA transporter (DAT) ratios with LID severity [15,16] and 5-HT neurons have been shown to mediate L-DOPA conversion and DA release during later stages of PD [17,18]. However, the lack of DA autoregulatory elements on 5-HT neurons can ultimately result in dysregulated DA efflux [19,20,21]. In corroboration, partial 5-HT lesions of the raphe nuclei greatly limited L-DOPA-induced increases in striatal DA release and the development of LID in hemi-parkinsonian rats [19,22,23].

Given the preclinical and clinical support implicating 5-HT neuroplasticity in LID, regulatory strategies that act at 5-HT_1A_ receptors (5-HT_1A_R), 5-HT_1B_ receptors (5-HT_1B_R), and the 5-HT transporter (SERT) have gained traction as therapeutic targets [24,25,26]. Intriguing retrospective clinical analyses have shown that the use of selective-serotonin-reuptake inhibitors (SSRIs) delayed LID onset and severity, implicating SERT as a therapeutic target [27]. Though the mechanisms by which SSRIs reduce LID remain speculative, there is evidence that SSRIs may normalize DA signaling in part by augmenting endogenous 5-HT levels at 5-HT_1A_R and 5-HT_1B_R [28,29,30]. In fact, agonists acting at 5-HT_1A_R, 5-HT_1B_R, or both, have shown promise preclinically and clinically, of which an extensive review has been done in our lab [25]. Unfortunately, many have been limited by their effects on L-DOPA efficacy, problematic side effect profiles, and induction of 5-HT syndrome [31,32,33,34].

This has led to efforts to combine these known anti-dyskinetic 5-HT actions by using multimodal 5-HT compounds, first developed for their anti-depressant actions. Recently, we confirmed the potential of dual-action 5-HT_1A/1B_R agonists and SERT blockers vilazodone (VZD) and vortioxetine (VTX) to dose-dependently attenuate LID [35]. The durability of this multimodal mechanism of action was demonstrated following chronic treatment with VZD, which sustained anti-dyskinetic efficacy while maintaining the motor benefits of L-DOPA [35,36,37]. Therefore, to further support the promise of such broader acting compounds, here, we used hemiparkinsonian rats to better characterize VTX. First, we sought to determine whether sub-chronic VTX administration maintains anti-dyskinetic efficacy without affecting L-DOPA’s benefits. We then examined the contribution of 5-HT_1A_R and 5-HT_1B_R to VTX’s anti-dyskinetic effects.

## 2. Materials and Methods

### 2.1. Animals

Adult male and female Sprague-Dawley rats were used for experimentation (N = 52; Envigo, Indianapolis, IN, USA). Animals were housed in plastic cages (22 cm high, 45 cm deep, and 23 cm wide) and had access to water and standardized lab chow ad libitum (Rodent Diet 5001; Lab Diet, Brentwood, MO, USA). The colony was maintained on a 12/12 h light/dark cycle beginning at 07:00 h at a room temperature of 22–23 °C. Animals were cared for in accordance with the Institutional Animal Care and Use Committee of Binghamton University and the “Guide for the Care and Use of Laboratory Animals” (Institute for Laboratory Animal Research, National Academic Press, Washington, DC, USA, 2011).

### 2.2. Surgeries

All animals underwent unilateral DA lesions using an infusion of 6-hydroxydopamine hydrobromide (6-OHDA; Sigma-Aldrich, St. Louis, MO, USA) into the left medial forebrain bundle (MFB) to produce a hemiparkinsonian phenotype. Following an injection of buprenorphine hydrochloride, (0.03 mg/kg, i.p.; Hospira Inc., Lake Forest, IL, USA) rats were anesthetized with inhalant isoflurane (2–3%; Sigma-Aldrich) in oxygen (2.5 L/min) and placed in a stereotaxic apparatus (David Kopf Instruments, Tujunga, CA, USA). After drilling a small hole in the skull, a 10 μL Hamilton syringe (Reno, NV) with a 26-gauge needle was lowered to the following coordinates with the incisor bar positioned at 5 mm below the interaural line: AP, −1.8 mm; ML, −2.0 mm; DV, −8.6 mm [38]. 6-OHDA was prepared at 3 μg/μL, dissolved in 0.9% NaCl + 0.1% ascorbic acid, and injected at a rate of 2 μL/min for a total volume of 4 μL. The needle was held at the target site for 5 min after infusion to allow for diffusion of toxin. Rats were monitored for 3 weeks post-surgery to ensure full recovery and lesion stabilization.

### 2.3. Pharmacological Treatments

For both experiments, sufficiently lesioned rats (n = 43) underwent 14 days of chronic treatment with levodopa methyl-ester (L-DOPA; 6 mg/kg, s.c.; Sigma-Aldrich, St. Louis, MO, USA) prepared in 0.9% NaCl + 0.1% ascorbic acid and co-administered with peripheral decarboxylase inhibitor benserazide hydrochloride (15 mg/kg; Alfa Aesar, Ward Hill, MA, USA), the combination hereafter referred to as L-DOPA, to elicit stable dyskinesia. Following the confirmation of stable dyskinesia, rats who met inclusion criteria (n = 35) later received acute or chronic injections of vortioxetine hydrobromide (VTX; Sigma-Aldrich) at a dose of 0, 3, or 10 mg/kg (s.c.) in a vehicle comprised of 25% dimethyl sulfoxide (DMSO; VWR International; Randor, PA, USA). Doses were chosen based on effective doses revealed in prior investigation [35]. All drugs were administered at 1 mL/kg.

### 2.4. Behavioral Assays and Procedures

#### 2.4.1. Forepaw Adjustment Stepping Test

The Forepaw Adjustment Stepping (FAS) test was employed to verify lesion efficacy and examine treatment-related improvements or subsequent reversal [29,34]. For this test, a trained experimenter holds each rat so that all but one forepaw are gently restrained. The free forepaw is dragged laterally on a surface at a rate of 90 cm/10 s, and steps are counted in the forehand and backhand directions for each paw in 3 separate trials. This test was chosen as both an exclusionary measure as well as a measure of treatment effects, as rats that perform poorly on this test typically show >80% striatal DA denervation [39].

#### 2.4.2. Abnormal Involuntary Movements Test

The Abnormal Involuntary Movements (AIMs) test was used to monitor the development of LID during chronic L-DOPA treatment and during pharmacological reversal testing. After L-DOPA injection, rats were placed in clear 20 cm × 45 cm plexiglass cylinders lined with bedding. A trained observer then measured the severity of LID according to the AIMs scale by quantifying the expression of axial, limb, and orolingual dyskinetic behaviors for 60 s every 10 min for 180 min beginning 10 min after L-DOPA injection [40]. “Axial” is defined as the uncontrolled torsion of the trunk contralateral to lesion. “Limb” is characterized by choreic and dystonic jerky movements of the limb contralateral to lesion. “Orolingual” is defined by rhythmic side-to-side jaw movements and tongue protrusions. Behaviors were ranked on a scale from 0 to 4, where 0 indicates the behavior was absent, 1 indicates the behavior was present for <30 s, 2 indicates the behavior was present for >30 s but <60 s, 3 indicates the behavior was present for the full 60 s, but could be interrupted by an external stimulus (tap on cylinder), and 4 indicates that the behavior was present for the full 60 s duration and could not be interrupted. Animals displaying ALO sums < 25 by day 14 of chronic L-DOPA treatment were excluded from further study, as an ALO score > 25 correlates with striatal DA loss exceeding 95% [41].

### 2.5. Neurochemical Analyses

#### 2.5.1. Tissue Preparation

Following completion of all testing, rats were allowed a washout period of at least 48 h before rapid decapitation and brain tissue collection. Whole brains were flash-frozen for 5–10 s in 2-methylbutane maintained at −80 °C (Sigma-Aldrich) before being stored at −80 °C. All brains were cut coronally on a cryostat (Lecia Biosystems; Wetzlar, Germany), and 2 mm diameter × 1 mm depth dorsal striatum tissue punches were collected +2.50 mm from bregma [38]. Tissue punches were stored at −80 °C until later analysis by high performance liquid chromatography (HPLC).

#### 2.5.2. HPLC Tissue Analysis

Lesions were confirmed in all rats post-mortem using reverse phase HPLC coupled with electrochemical detection to evaluate striatal levels of 3,4-dihydroxyphenylacetic acid (DOPAC), DA, 5-hydroxyindoleacetic acid (5-HIAA), and 5-HT. Tissue samples were homogenized in ice-cold 0.1 M perchloric acid containing 10 mM EDTA and 1% ethanol before being spun for 45 min at 14,000 rpm while being maintained at 4 °C. Supernatant was separated and loaded into an AMUZA AS-700 autosampler (San Diego, CA, USA) and continuously maintained at 4 °C. Mobile phase comprised of 0.1 M citrate-acetate buffer, including 17% methanol, 190 mg/L sodium octansulfonate, and 5 mg/L EDTA-2Na, adjusted to pH 3.5, was propelled by an EICOM HTEC-510 (AMUZA) at a flow rate of 500 μL/min. Samples were chromatographically separated by a reverse-phase separation column (EICOMPAK SC-5ODS, 3.0 id × 100 mm; AMUZA) held at 25 °C, then samples were passed through a graphite working electrode (WE-3G; AMUZA) at an applied potential of +750 mV vs. Ag/AgCl with a time constant of 3.0 s. Neurotransmitters were oxidized upon contact with the graphite electrode of the electrochemical detection cell, generating a spike in measured current. This measure of electrical current over time was recorded and later analyzed using Clarity software (version 8.7.; Data Apex; Tucson, AZ, USA). Each neurotransmitter was detected as a peak; therefore, peak areas were converted to ng per mg of tissue using a standard curve (1E-6 M to 1E-10 M).

### 2.6. Statistical Analysis

All AIMs data (expressed as median + median absolute deviation; M.A.D.) were assessed using nonparametric statistics. Between-subjects designs were assessed using a Kruskal–Wallis ANOVA followed by Dunn post hoc analyses to examine treatment effects. Within-subjects designs were assessed using a Friedman’s ANOVA followed by a Wilcoxon post hoc analyses of treatment effects. All FAS data (expressed as mean percent intact + standard error of the mean; S.E.M.) were assessed using repeated measures ANOVAs followed by Fisher LSD post hoc analyses as appropriate. All HPLC monoamine data collected from tissue were analyzed by *t*-tests.

### 2.7. Experimental Design

#### 2.7.1. Experiment 1: Sub-Chronic Vortioxetine Intervention in Dyskinetic Rats

**Figure 1:**
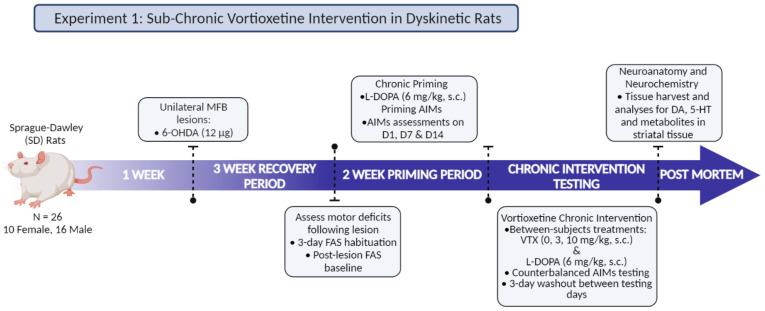
Experimental Design 1: Chronic Vortioxetine Intervention in Dyskinetic Rats.

As detailed in Figure 1, male and female Sprague-Dawley rats (N = 26; 10F, 16M) underwent unilateral 6-OHDA MFB lesion surgeries after 1 week of acclimation and 5 days of handling. After surgery, rats were allowed 3 weeks for recovery before undergoing baseline FAS testing and then 2 weeks of daily L-DOPA treatment. AIMs were assessed on days 1, 7, and 14 to determine AIMs development (criterion for inclusion: ALO sum > 25) and create equally dyskinetic groups. Following priming, rats began 2 weeks of daily VTX (0, 3 or 10 mg/kg, s.c.) and L-DOPA (6 mg/kg) treatment in a between-subjects design. VTX was administered 5 min prior to L-DOPA. Sixty min into the AIMs test on days 15, 22, and 29, the FAS test was again employed to assess drug-induced changes in motor performance. After a washout of at least 2–3 days, rats were euthanized via rapid decapitation and brain tissue was collected for later HPLC analyses.

#### 2.7.2. Experiment 3: Role of 5-HT_1A_ and 5-HT_1B_ Receptors in Vortioxetine’s Behavioral Effects

**Figure 2:**
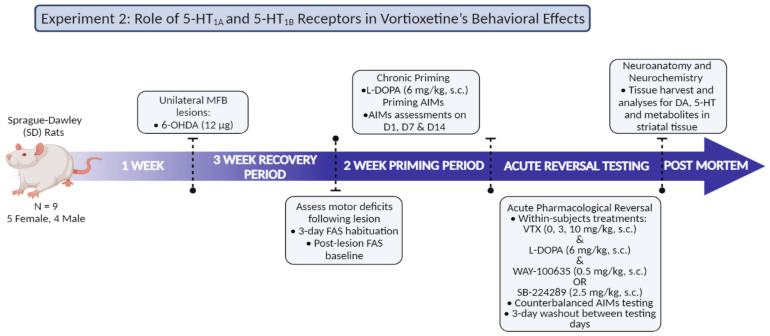
Experimental Design 2: Role of 5-HT_1A_ and 5-HT_1B_ Receptors in Vortioxetine’s Behavioral Effects.

As shown in Figure 2, to determine the contribution of 5-HT_1_R to VTX effects, 5-HT_1A_ or 5-HT_1B_ antagonists were used. For this experiment, Sprague-Dawley rats (N = 9, 5F, 4M) were rendered hemiparkinsonian via 6-OHDA left MFB lesioning. Following post-operative recovery, rats underwent baseline FAS testing to verify lesion severity, followed by 2 weeks of daily L-DOPA (6 mg/kg). Rats were assessed for AIMs on days 1, 7, and 14 to monitor LID progression. Thereafter, rats meeting criterion (ALO AIMs > 25) underwent a within-subjects, counterbalanced treatment paradigm, where all rats received both L-DOPA (6 mg/kg) and VTX (0, 3, and 10 mg/kg), as well as 5-HT_1A_ antagonist WAY-100635 (0, 0.5 mg/kg) or 5-HT_1B_ antagonist SB-224289 (0, 2.5 mg/kg) and AIMs and FAS assessments. Within a week of final testing, rats were euthanized via rapid decapitation, and HPLC was performed on striatal tissue.

## 3. Results

### 3.1. Development of L-DOPA-Induced AIMs across Experiments

To monitor the development of LID, all rats received 2 weeks of daily treatment with L-DOPA prior to sub-chronic VTX intervention in Experiment 1 (N = 26), or for 5-HT pharmacological studies in Experiment 2 (N = 9). Of the rats that entered the treatment phase, 35 of 43 met inclusion criterion (ALO Sum > 25 by day 14). In Experiment 1, non-parametric Friedman’s ANOVAs demonstrated a significant effect of day (χ^2^(2) = 39, *p* < 0.05). Post hoc analyses revealed that ALO AIMs were elevated within a week of starting treatment compared to Day 1 and were maintained when tested a week later (both *p* < 0.05). When analyzing the effects of daily L-DOPA on ALO AIMs in Experiment 2, the same pattern of effects was observed (χ^2^(2) = 17.543, *p* < 0.05), supporting prior work that within 1 week of daily treatment, AIMs stabilize at this dose of L-DOPA [35,36,42].

### 3.2. Experiment 1: Sub-Chronic Vortioxetine Intervention in Dyskinetic Rats

**Figure 3:**
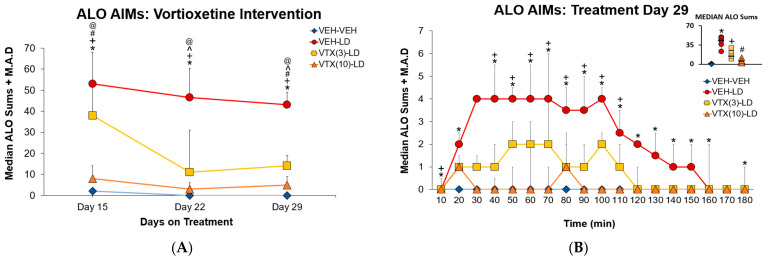
Experiment 1: Sub-Chronic Vortioxetine Intervention in Dyskinetic Rats. Rats (N = 26) were treated with vortioxetine (VTX; 3, 10 mg/kg, s.c.) or vehicle (VEH) 5 min prior to administration of L-DOPA (LD; 6 mg/kg, s.c.) or VEH for 14 consecutive days following a 2 week daily L-DOPA only regimen in a between-subjects design (n = 6 VEH-VEH, n = 6 VEH-LD; n = 7 VTX(3)-LD; n = 7 VTX(10)-LD). Axial, limb, and orolingual (ALO) abnormal involuntary movements were observed over a 3 h time course every 10 min following the L-DOPA injection. To determine effects over the 2-week testing period, (**A**) weekly ALO AIMs sums are shown and were expressed as median and mean absolute deviation (M.A.D.). To examine the time-course of effects on the final day of testing (**B**), individual timepoints are shown for each group’s ALO AIMs. Significant differences in ALO AIMs between treatments on each day (**A**) and across time course (**B**) were determined using non-parametric Kruskal–Wallis ANOVAs with Mann–Whitney U’s post hoc tests. * *p* < 0.05 VEH-VEH vs. VEH-LD; + *p* < 0.05 VEH-VEH vs. VTX(3)-LD; # *p* < 0.05 VEH-VEH vs. VTX(10)-LD; ^ *p* < 0.05 VEH-LD vs. VTX(3); @ *p* < 0.05 VEH-LD vs. VTX(10)-LD.

With the purpose of understanding the anti-dyskinetic benefits of sub-chronic VTX intervention over time, analyses revealed significant treatment effects on each day (χ^2^(3) = 21.582, *p* < 0.05). As shown in Figure 3, post hoc analyses demonstrated differences between VEH-VEH and all other treatments on Day 15 and Day 29 (all *p* < 0.05; Figure 3A). On Day 22, there was a significant difference in ALO median sums between vehicle treatment (VEH-VEH) versus all treatments except 10 mg/kg of VTX with L-DOPA (*p* < 0.05; Figure 3A). Moreover, pretreatment of 3 vs. 10 mg/kg VTX produced statistically unique effects on all 3 treatment days as well. For example, the high dose of VTX reduced AIMs severity in comparison to L-DOPA treatment on all treatment days (*p* < 0.05; Figure 3A), whereas the low dose of VTX was without significant effects on day 15. Figure 3B displays the within session time-course effects of sub-chronic VTX on ALO AIMs on Day 29. Kruskal–Wallis analyses revealed that the 10 mg/kg dose suppressed dyskinesia from timepoint 30–150 min while 3 mg/kg reduced it from timepoint 50–110 min (all *p* < 0.05).

**Figure 4:**
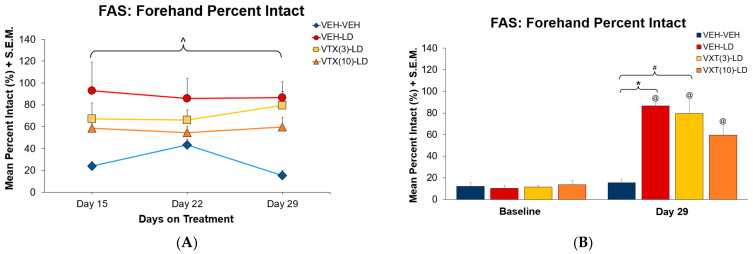
Experiment 1: Effects of Chronic Vortioxetine Intervention on Forehand Motor Performance. Rats (N = 26) were treated with vortioxetine (VTX; 3, 10 mg/kg, s.c.) or vehicle (VEH) 5 min prior to administration of L-DOPA (LD; 6 mg/kg, s.c.) or VEH (n = 6 VEH-VEH; n = 6 VEH-LD; n = 7 VTX(3)-LD; n = 7 VTX(10)-LD). At 60 min into each abnormal involuntary movements (AIMs) session, the forepaw adjusting steps (FAS) test was employed to evaluate motor performance. Forehand motor performance was expressed as forehand percent intact values ([number of lesioned forepaw steps/number of non-lesioned forepaw steps] × 100). FAS data is shown as mean percent intact and standard error mean (S.E.M.). Analysis of FAS measures across the 2 week period (**A**) were determined using 3 × 4 mixed-model ANOVAs. ^ *p* < 0.05 VEH-LD and VTX(3)-LD vs. VEH-VEH. Final day 29 testing (**B**) were determined using 2 × 4 mixed-model ANOVAs. * *p* < 0.05 VEH-VEH vs. VEH-LD; # *p* < 0.05 VEH-VEH vs. VTX(3)-LD; @ *p* < 0.05 Any treatment vs. Baseline.

Analyses of the effects of VTX intervention on L-DOPA improvement of stepping over time (Figure 4A) revealed a significant main effect of treatment (F(1,4) = 5.10, *p* < 0.05) but no interaction between day and treatment. Post hoc analyses of the main effect revealed significant increases in stepping between VEH-VEH vs. VEH-LD, VTX(3)-LD, and VTX(10-LD across days (*p* < 0.05). In Figure 4B, a comparison of baseline stepping vs. that on day 29 revealed a main effect of day (F(1) = 149.282, *p* < 0.05). Selected pairwise comparisons across time demonstrated that any pretreatment paired with L-DOPA on day 29 increased stepping versus baseline (all *p* < 0.05) while significant stepping improvements due to treatment were observed in VEH-LD and VTX(3)-LD in comparison to VEH-VEH (*p* < 0.05).

### 3.3. Experiment 2: Role of 5-HT_1A_ and 5-HT_1B_ Receptors in Vortioxetine’s Behavioral Effects

**Figure 5:**
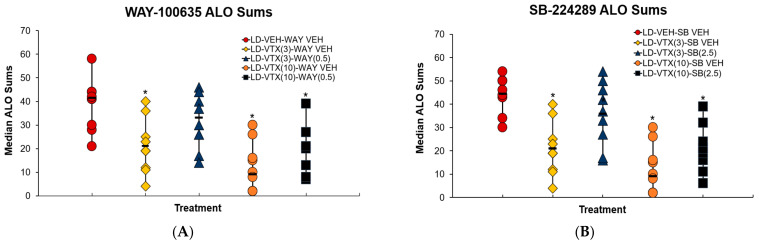
Experiment 2: Role of 5-HT_1A_ and 5-HT_1B_ Receptors in Vortioxetine’s Behavioral Effects. In a within-subjects counterbalanced design, rats (N = 9, 4M, 5F) underwent 14 days of chronic L-DOPA (6 mg/kg, s.c.) priming to establish profound dyskinesia. Following priming, rats were treated with the 5-HT_1A_ antagonist WAY-100635 (WAY) or WAY vehicle (WAY VEH), or the 5-HT_1B_ antagonist SB-224289 (SB; 2.5 mg/kg; s.c.) or SB vehicle (SB VEH; s.c.), 5 min prior to the administration of vortioxetine (VTX; 3, 10 mg/kg, s.c.) or VTX vehicle (VEH). Rats were then administered L-DOPA (6 mg/kg, s.c.) 5 min after VTX/VTX VEH treatment and were subjected to 180 min AIMs sessions and FAS. (**A**,**B**) AIMs axial, limb, and orolingual behaviors (ALO) were observed over a 3 h time course every 10 min following the L-DOPA injection with the administration of WAY (**A**) or SB (**B**). Significant differences in ALO SUMs between treatments were determined using non-parametric Kruskal–Wallis ANOVAs with Mann-Whitney U’s post hoc tests. * *p* < 0.05 Any treatment vs. LDVEHVEH.

To determine the contribution of 5-HT receptor subtypes on VTX’s behavioral effects, the selective 5-HT_1A_R and 5-HT_1B_R antagonists WAY-100635 (WAY) and SB-224289 (SB) were used. Friedman’s ANOVA analyses demonstrated main effects of treatment for each (WAY χ^2^(4) = 34.083, *p* < 0.05; SB χ^2^(4) = 32.163, *p* < 0.05). Post hoc analyses across treatments demonstrated that as in the other experiments both doses of VTX reduced ALO AIMs in a dose-dependent manner (all *p* < 0.05; Figure 5A,B). Moreover, blocking 5-HT_1A_R or 5-HT_1B_R reversed the effects of the lower dose of VTX (both *p* < 0.05; Figure 5A,B). Interestingly, in neither WAY nor SB alone were able to fully reverse the effects of higher dose VTX, the combinations of which remained lower than L-DOPA alone (both *p* < 0.05).

### 3.4. High Performance Liquid Chromatography

In order to assess relative DA, 5-HT and respective metabolite concentrations within striatal tissue, HPLC was used. Following paired-samples *t*-tests, HPLC results indicated significant reductions in concentrations of DOPAC (t(34) = 12.43, *p* < 0.05), DA (t(34) = 9.09, *p* < 0.05), DA turnover (DOPAC/DA; t(34) = 1.975, *p* < 0.05), 5-HIAA (t(34) = 10.76, *p* < 0.05), 5-HT (t(34) = 2.45, *p* < 0.05), and 5-HT turnover (5-HIAA/5-HT; t(34) = 9.74, *p* < 0.05) within lesioned striata when compared to intact striata (Table 1), indicating sufficient lesioning across all animals in both experiments 1 and 2.

## 4. Discussion

Across many studies and laboratories, researchers have consistently found that neuroplasticity within the 5-HT system of the parkinsonian brain provides potential targets for preventing and managing LID [25,43]. While several candidates have been proposed and tested, many mitigate LID at the expense of L-DOPA’s efficacy or produce intrinsic side effects limiting clinical translation [31,32,34,44].

In recent years, in attempts to overcome prior limitations, several novel strategies have been pursued, including biased and selective 5-HT_1A_R agonists [45,46], combination strategies [42,47], and multimodal 5-HT approaches [35,36,48]. With several studies now supporting a combined SERT blockade and 5-HT_1_R agonism as a promising profile, we sought to characterize the durability and pharmacology of the FDA-approved multimodal 5-HT antidepressant VTX in the hemiparkinsonian rodent.

In Experiment 1, the effects of chronic VTX intervention on LID and motor performance were examined. We found AIMs severity was reduced in a dose-dependent manner (Figure 3). Within a week of treatment, both low and high doses of VTX reduced AIMs by >75%, and this effect stabilized into day 29 (Figure 3A). On the final day of treatment, 10mg/kg of VTX showed the greatest reduction of LID expression, although both doses of VTX resulted in lower ALO sums than the VTX vehicle. Given the burgeoning efficacy of the 3 mg/kg dose, perhaps even lower doses, over time, may be effective. Regarding motor performance, there were no observed interactions between treatment and day on motor performance over the 2 weeks (Figure 4). FAS data revealed improved stepping compared to baseline for both VTX doses paired with L-DOPA, indicating a reversal and maintenance in lesion-induced motor deficits (Figure 4). The reduction in AIMs and improvement in stepping compared to baseline at both doses of VTX has been observed with acute administration in our lab [35]. On day 29, there was a significant difference in stepping between VEH-VEH compared to VEH-LD and the low dose of VTX, with the higher dose being intermediate (Figure 4), suggesting possible suppression of L-DOPA efficacy at higher doses of VTX. This would need to be taken into consideration, as patients often prefer greater mobility associated with LID as opposed to immobility without LID [49].

VTX’s pharmacological profile implicates clear actions at 5-HT_1_R [50,51,52]. Thus, to investigate the receptor contributions of VTX in alleviating LID, experiment 2 employed 5-HT_1A_R (WAY-100635) and 5-HT_1B_R (SB-224289) antagonists. Following either 5-HT_1A_R or 5-HT_1B_R blockade, the effects of VTX were partially reversed relative to dose (Figure 5A,B). Collectively, these findings corroborate prior work supporting 5-HT_1A_R and 5-HT_1B_R individually or together as a therapeutic agent for the treatment of LID [25,30,53,54,55]. Interestingly, neither alone were sufficient to fully reverse VTX effects, suggesting activity at both receptor subtypes may be needed to produce maximal effects or other VTX mechanisms also contribute.

Indeed, as a multimodal drug, VTX also targets 5-HT_1D_, 5-HT_3_, and 5-HT_7_ receptors, as well as SERT [56,57], though these actions are species-specific. The affinity for 5-HT_1A_ and 5-HT_7_ are higher in humans versus rats [51], with a notable preferential affinity for 5-HT_1A_R in humans (K_i_ = 15 nM) compared to rats (K_i_ = 230 nM). Conversely VTX also acts as a partial agonist at the 5-HT_1B_R to a greater extent in rats (K_i_ = 16 nM) than in humans (K_i_ = 33 nM) while it is potently antagonistic at 5-HT_3_ for both rats (K_i_ = 1.1 nM) and humans (K_i_ = 3.7 nM; [50]). VTX has also been shown to display moderate antagonism at 5-HT_7_ in humans (K_i_ = 19 nM) and to a much lesser extent in rats (K_i_ = 200 nM; [50,58]). VTX binds to SERT with a potent affinity in humans (K_i_ = 1.6 nM) and to a lesser extent in rats (K_i_ = 8.6 nM; [50]).

Not all of VTX’s targets have been investigated for actions against LID, but there are hints of the involvement of some. For example, the 5-HT_3_ receptor antagonist ondansetron displayed anti-dyskinetic effects in hemiparkinsonian rats [59,60]. Given prior work that SERT blockade reduces LID [28,29,33], an additional intriguing feature of VTX is that its antagonism of 5-HT_7_ receptors may potentiate the effects of SERT inhibition [57,61].

Finally, there is increasing evidence that VTX’s broad actions can modulate known LID mechanisms both pre- and post-synaptically. Pre-synaptically, 5-HT_1_R agonists likely reduce LID in part by the presynaptic inhibition and attenuation of DA release from serotonergic neurons [17,24,62]. There is also evidence that coincident activation of 5-HT_1A_ heteroreceptors on cortico-striatal projection neurons reduce striatal glutamate release, contributing to decreased transmission and increased anti-dyskinetic effects [63,64]. Interestingly, the combination of 5-HT_1_R compounds 8-OH-DPAT and CP-94253 with targeted actions at both 5-HT_1A_R and 5-HT_1B_R, respectively, significantly reduced LID at sub-threshold doses [19,30,65]. Post-synaptically, 5-HT_1_R agonists reduce known immediate early genes and pro-dyskinetic transcripts in direct pathway D1 receptor expressing striatal neurons [30,36,37,53,66]. Thus, VTX’s multimodal actions at both 5-HT_1A_R and 5-HT_1B_R may account for its promising anti-dyskinetic effects seen even at the low dose (3mg/kg), in part through articulation across LID neurocircuitry.

In conclusion, VTX durably and dose-dependently reduced dyskinesia following L-DOPA administration without reducing L-DOPA’s motor efficacy. Additionally, VTX’s anti-dyskinetic effects were carried in part by its actions at both 5-HT_1A_R and 5-HT_1B_R. Ultimately, these findings both emphasize and elucidate potential mechanisms underlying VTX’s promising utility as an anti-dyskinetic agent in the treatment of LID and support its development as a safe and readily repositionable clinical pharmacotherapy.

## Figures and Tables

**Table 1:** High Performance Liquid Chromatography.

Side	DOPAC (ng/mg)	DA (ng/mg)	DOPAC/DA	5-HIAA (ng/mg)	5-HT (ng/mg)	5-HIAA/5-HT
Intact (right)	2.06 ± 0.15	9.74 ± 1.07	0.28 ± 0.03	0.69 ± 0.06	0.11 ± 0.01	7.49 ± 0.69
Lesion (left)	0.12 ± 0.01 *	0.09 ± 0.01 *	2.31 ± 0.27 *	0.05 ± 0.01 *	0.08 ± 0.01 *	1.02 ± 0.23 *

Effects of 6-hydroxydopamine lesion on concentrations of monoamine and metabolite levels and turnover ratios in intact and lesioned striata. 3-4-dihydroxyphenylacetic acid (DOPAC); dopamine (DA); 5-hydroxyindoleacetic acid (5-HIAA); serotonin (5-HT). Units are nanogram of monoamine or metabolite per milligram of tissue or ratios of metabolite to monoamine (mean ± S.E.M.) with percentage of vehicle group in parentheses. Differences between group means were determined with paired *t*-tests. * *p* < 0.05 compared with intact (right) striata.

## Data Availability

The data presented in this study are available on request from the corresponding author.

## References

[1] Jankovic J. (2008). Parkinson’s disease: Clinical features and diagnosis. J. Neurol. Neurosurg. Psychiatry.

[2] Davie C.A. (2008). A review of Parkinson’s disease. Br. Med. Bull..

[3] Ehringer H., Hornykiewicz O. (1998). Distribution of noradrenaline and dopamine (3-hydroxytyramine) in the human brain and their behavior in diseases of the extrapyramidal system. Park. Relat. Disord..

[4] Dorsey E.R., Elbaz A., Nichols E., Abbasi N., Abd-Allah F., Abdelalim A., Adsuar J.C., Ansha M.G., Choi J.-Y.J., Collado-Mateo D. (2018). Global, regional, and national burden of Parkinson’s disease, 1990–2016: A systematic analysis for the Global Burden of Disease Study 2016. Lancet Neurol..

[5] Smith Y., Wichmann T., Factor S.A., DeLong M.R. (2012). Parkinson’s disease therapeutics: New developments and challenges since the introduction of levodopa. Neuropsychopharmacology.

[6] Ahlskog J.E., Muenter M.D. (2001). Frequency of levodopa-related dyskinesias and motor fluctuations as estimated from the cumulative literature. Mov. Disord..

[7] Connolly B.S., Lang A.E. (2014). Pharmacological treatment of Parkinson disease: A review. JAMA.

[8] Chapuis S., Ouchchane L., Metz O., Gerbaud L., Durif F. (2005). Impact of the motor complications of Parkinson’s disease on the quality of life. Mov. Disord..

[9] Suh D.-C., Pahwa R., Mallya U. (2012). Treatment patterns and associated costs with Parkinson’s disease levodopa induced dyskinesia. J. Neurol. Sci..

[10] Péchevis M., Clarke C.E., Vieregge P., Khoshnood B., Deschaseaux-Voinet C., Berdeaux G., Ziegler M., Trial Study Group (2005). Effects of dyskinesias in Parkinson’s disease on quality of life and health-related costs: A prospective European study. Eur. J. Neurol..

[11] Rylander D., Parent M., O’Sullivan S.S., Dovero S., Lees A.J., Bezard E., Descarries L., Cenci M.A. (2010). Maladaptive plasticity of serotonin axon terminals in levodopa-induced dyskinesia. Ann. Neurol..

[12] Pagano G., Niccolini F., Fusar-Poli P., Politis M. (2017). Serotonin transporter in Parkinson’s disease: A meta-analysis of positron emission tomography studies. Ann. Neurol..

[13] Maeda T., Nagata K., Yoshida Y., Kannari K. (2005). Serotonergic hyperinnervation into the dopaminergic denervated striatum compensates for dopamine conversion from exogenously administered l-DOPA. Brain Res..

[14] Zeng B.Y., Iravani M.M., Jackson M.J., Rose S., Parent A., Jenner P. (2010). Morphological changes in serotoninergic neurites in the striatum and globus pallidus in levodopa primed MPTP treated common marmosets with dyskinesia. Neurobiol. Dis..

[15] Lee J.Y., Seo S., Lee J.S., Kim H.J., Kim Y.K., Jeon B.S. (2015). Putaminal serotonergic innervation: Monitoring dyskinesia risk in Parkinson disease. Neurology.

[16] Roussakis A.A., Politis M., Towey D., Piccini P. (2016). Serotonin-to-dopamine transporter ratios in Parkinson disease: Relevance for dyskinesias. Neurology.

[17] Politis M., Wu K., Loane C., Brooks D.J., Kiferle L., Turkheimer F.E., Bain P., Molloy S., Piccini P. (2014). Serotonergic mechanisms responsible for levodopa-induced dyskinesias in Parkinson’s disease patients. J. Clin. Investig..

[18] Nishijima H., Tomiyama M. (2016). What mechanisms are responsible for the reuptake of levodopa-derived dopamine in Parkinsonian striatum?. Front. Neurosci..

[19] Carta M., Carlsson T., Kirik D., Björklund A. (2007). Dopamine released from 5-HT terminals is the cause of L-DOPA-induced dyskinesia in parkinsonian rats. Brain.

[20] Carta M., Carlsson T., Muñoz A., Kirik D., Björklund A. (2008). Serotonin–dopamine interaction in the induction and maintenance of L-DOPA-induced dyskinesias. Prog. Brain Res..

[21] Sellnow R.C., Newman J.H., Chambers N., West A.R., Steece-Collier K., Sandoval I.M., Benskey M.J., Bishop V., Manfredsson F. (2019). Regulation of dopamine neurotransmission from serotonergic neurons by ectopic expression of the dopamine D2 autoreceptor blocks levodopa-induced dyskinesia. Acta Neuropathol. Commun..

[22] Tanaka H., Kannari K., Maeda T., Tomiyama M., Suda T., Matsunaga M. (1999). Role of 5-HT neurons in L-DOPA-derived extracellular dopamine in the striatum of 6-OHDA-lesioned rats. NeuroReport.

[23] Eskow K.L., Dupre K.B., Barnum C.J., Dickinson S.O., Park J.Y., Bishop C. (2009). The role of the dorsal raphe nucleus in the development, expression, and treatment of L-dopa-induced dyskinesia in hemiparkinsonian rats. Synapse.

[24] Carta M., Tronci E. (2014). Serotonin system implication in l-DOPA-induced dyskinesia: From animal models to clinical investigations. Front. Neurol..

[25] Lanza K., Bishop C. (2018). Serotonergic targets for the treatment of L-DOPA-induced dyskinesia. J. Neural Transm..

[26] Farajdokht F., Sadigh-Eteghad S., Majdi A., Pashazadeh F., Vatandoust S.M., Ziaee M., Safari F., Karimi P., Mahmoudi J. (2020). Serotonergic system modulation holds promise for L-DOPA-induced dyskinesias in hemiparkinsonian rats: A systematic review. EXCLI J..

[27] Mazzucchi S., Frosini D., Ripoli A., Nicoletti V., Linsalata G., Bonuccelli U., Ceravolo R. (2014). Serotonergic antidepressant drugs and L-dopa-induced dyskinesias in Parkinson’s disease. Acta Neurol. Scand..

[28] Bishop C., George J.A., Buchta W., Goldenberg A.A., Mohamed M., Dickinson S.O., Eissa S., Eskow Jaunarajs K.L. (2012). 5-HT transporter inhibition attenuates l-DOPA-induced dyskinesia without compromising l-DOPA efficacy in hemi-parkinsonian rats. Eur. J. Neurosci..

[29] Conti M.M., Ostock C.Y., Lindenbach D., Goldenberg A.A., Kampton E., Dell’isola R., Katzman A.C., Bishop C. (2014). Effects of prolonged selective serotonin reuptake inhibition on the development and expression of L-DOPA-induced dyskinesia in hemi-parkinsonian rats. Neuropharmacology.

[30] Munoz A., Li Q., Gardoni F., Marcello E., Qin C., Carlsson T., Kirik D., Di Luca M., Björklund A., Bezard E. (2008). Combined 5-HT1A and 5-HT1B receptor agonists for the treatment of L-DOPA-induced dyskinesia. Brain.

[31] Chung K.A., Carlson N.E., Nutt J.G. (2005). Short-term paroxetine treatment does not alter the motor response to levodopa in PD. Neurology.

[32] Goetz C.G., Damier P., Hicking C., Laska E., Müller T., Olanow C.W., Rascol O., Russ H. (2007). Sarizotan as a treatment for dyskinesias in Parkinson’s disease: A double-blind placebo-controlled trial. Mov. Disord..

[33] Fidalgo C., Ko W.K., Tronci E., Li Q., Stancampiano R., Chuan Q., Bezard E., Carta M. (2015). Effect of serotonin transporter blockade on L-DOPA-induced dyskinesia in animal models of Parkinson’s disease. Neuroscience.

[34] Lindenbach D., Palumbo N., Ostock C.Y., Vilceus N., Conti M.M., Bishop C. (2015). Side effect profile of 5-HT treatments for Parkinson’s disease and L-DOPA-induced dyskinesia in rats. Br. J. Pharmacol..

[35] Smith S., Sergio J., Coyle M., Elder K., Centner A., Cohen S., Terry M., Lipari N., Glinski J., Wheelis E. (2022). The effects of Vilazodone, YL-0919 and Vortioxetine in hemiparkinsonian rats. Psychopharmacology.

[36] Meadows S.M., Chambers N.E., Conti M.M., Bossert S.C., Tasber C., Sheena E., Varney M., Newman-Tancredi A., Bishop C. (2017). Characterizing the differential roles of striatal 5-HT1A auto- and hetero-receptors in the reduction of l-DOPA-induced dyskinesia. Exp. Neurol..

[37] Altwal F., Moon C., West A.R., Steiner H. (2020). The multimodal serotonergic agent Vilazodone inhibits L-DOPA-induced gene regulation in striatal projection neurons and associated dyskinesia in an animal model of Parkinson’s disease. Cells.

[38] Paxinos G., Watson C. (1998). The Rat Brain in Stereotaxic Coordinates.

[39] Chang J., Wachtel S., Young D., Kang U. (1999). Biochemical and anatomical characterization of forepaw adjusting steps in rat models of Parkinson’s disease: Studies on medial forebrain bundle and striatal lesions. Neuroscience.

[40] Lundblad M., Andersson M., Winkler C., Kirik D., Wierup N., Cenci M.A. (2002). Pharmacological validation of behavioural measures of akinesia and dyskinesia in a rat model of Parkinson’s disease. Eur. J. Neurosci..

[41] Taylor J.L., Bishop C., Walker P.D. (2005). Dopamine D1 and D2 receptor contributions to L-DOPA-induced dyskinesia in the dopamine-depleted rat. Pharmacol. Biochem. Behav..

[42] Cohen S.R., Terry M.L., Coyle M., Wheelis E., Centner A., Smith S., Glinski J., Lipari N., Budrow C., Manfredsson F.P. (2022). The multimodal serotonin compound Vilazodone alone, but not combined with the glutamate antagonist Amantadine, reduces L-dopa induced dyskinesia in hemiparkinsonian rats. Pharmacol. Biochem. Behav..

[43] Corsi S., Stancampiano R., Carta M. (2021). Serotonin/dopamine interaction in the induction and maintenance of L-DOPA-induced dyskinesia: An update. Prog. Brain Res..

[44] Kannari K., Kurahashi K., Tomiyama M., Maeda T., Arai A., Baba M., Suda T., Matsunaga M. (2002). Tandospirone citrate, a selective 5-HT1A agonist, alleviates L-DOPA-induced dyskinesia in patients with Parkinson’s disease. No Shinkei.

[45] Chambers N.E., Meadows S.M., Taylor A., Sheena E., Lanza K., Conti M.M., Bishop C. (2019). Effects of muscarinic acetylcholine m1 and m4 receptor blockade on dyskinesia in the hemi-parkinsonian rat. Neuroscience.

[46] Depoortere R., Johnston T.H., Fox S.H., Brotchie J.M., Newman-Tancredi A. (2020). The selective 5-HT_1A_ receptor agonist, NLX-112, exerts anti-dyskinetic effects in MPTP-treated macaques. Park. Relat. Disord..

[47] Pinna A., Ko W.K.D., Costa G., Tronci E., Fidalgo C., Simola N., Li Q., Tabrizi M.A., Bezard E., Carta M. (2016). Antidyskinetic effect of A2A and 5HT1A/1B receptor ligands in two animal models of Parkinson’s disease. Mov. Disord..

[48] Tronci E., Fidalgo C., Stancampiano R., Carta M. (2015). Effect of selective and non-selective serotonin receptor activation on l-DOPA-induced therapeutic efficacy and dyskinesia in parkinsonian rats. Behav. Brain Res..

[49] Thanvi B., Lo N., Robinson T. (2007). Levodopa-induced dyskinesia in Parkinson’s disease: Clinical features, pathogenesis, prevention and treatment. Postgrad. Med. J..

[50] Sanchez C., Asin K.E., Artigas F. (2015). Vortioxetine, a novel antidepressant with multimodal activity: Review of preclinical and clinical data. Pharmacol. Ther..

[51] Chen G., Højer A.M., Areberg J., Nomikos G. (2018). Vortioxetine: Clinical pharmacokinetics and drug interactions. Clin. Pharmacokinet..

[52] Blier P., Ward N.M. (2003). Is there a role for 5-HT1A agonists in the treatment of depression?. Biol. Psychiatry.

[53] Bishop C., Krolewski D.M., Eskow K.L., Barnum C.J., Dupre K.B., Deak T., Walker P.D. (2009). Contribution of the striatum to the effects of 5-HT1A receptor stimulation in L-DOPA-treated hemiparkinsonian rats. J. Neurosci. Res..

[54] Jaunarajs K.L.E., Dupre K.B., Steiniger A., Klioueva A., Moore A., Kelly C., Bishop C. (2009). Serotonin 1B receptor stimulation reduces D1 receptor agonist-induced dyskinesia. Neuroreport.

[55] Bezard E., Tronci E., Pioli E.Y., Li Q., Porras G., Björklund A., Carta M. (2013). Study of the antidyskinetic effect of eltoprazine in animal models of levodopa-induced dyskinesia. Mov. Disord..

[56] Stahl S.M. (2015). Modes and nodes explain the mechanism of action of vortioxetine, a multimodal agent (MMA): Enhancing serotonin release by combining serotonin (5HT) transporter inhibition with actions at 5HT receptors (5HT1A, 5HT1B, 5HT1D, 5HT7 receptors). CNS Spectr..

[57] D’Agostino A., English C.D., Rey J.A. (2015). Vortioxetine (Brintellix): A new serotonergic antidepressant. Pharm. Ther..

[58] Álvarez F.J. (2016). Parkinson’s disease, antiparkinson medicines, and driving. Expert Rev. Neurother..

[59] Aboulghasemi N., Jahromy M.H., Ghasemi A. (2019). Anti-dyskinetic efficacy of 5-HT3 receptor antagonist in the hemi-parkinsonian rat model. IBRO Rep..

[60] Zoldan J., Friedberg G., Livneh M., Melamed E. (1995). Psychosis in advanced Parkinson’s disease: Treatment with ondansetron, a 5-HT3 receptor antagonist. Neurology.

[61] Stenkrona P., Halldin C., Lundberg J. (2013). 5-HTT and 5-HT(1A) receptor occupancy of the novel substance vortioxetine (Lu AA21004). A PET study in control subjects. Eur. Neuropsychopharmacol..

[62] Politis M., Wu K., Loane C., Quinn N.P., Brooks D.J., Rehncrona S., Bjorklund A., Lindval O., Piccini P. (2010). Serotonergic neurons mediate dyskinesia side effects in Parkinson’s patients with neural transplants. Sci. Transl. Med..

[63] Mignon L.J., Wolf W.A. (2005). 8-hydroxy-2-(di-n-propylamino) tetralin reduces striatal glutamate in an animal model of Parkinson’s disease. Neuroreport.

[64] Dupre K.B., Eskow K.L., Barnum C.J., Bishop C. (2008). Striatal 5-HT1A receptor stimulation reduces D1 receptor-induced dyskinesia and improves movement in the hemiparkinsonian rat. Neuropharmacology.

[65] Muñoz A., Carlsson T., Tronci E., Kirik D., Björklund A., Carta M. (2009). Serotonin neuron-dependent and-independent reduction of dyskinesia by 5-HT1A and 5-HT1B receptor agonists in the rat Parkinson model. Exp. Neurol..

[66] Tomiyama M., Kimura T., Maeda T., Kannari K., Matsunaga M., Baba M. (2005). A serotonin 5-HT1A receptor agonist prevents behavioral sensitization to L-DOPA in a rodent model of Parkinson’s disease. Neurosci. Res..

